# A comparative study of biological and metabolic biomarkers between healthy individuals and patients with acne vulgaris: A cross-sectional study protocol

**DOI:** 10.1097/MD.0000000000008554

**Published:** 2017-11-10

**Authors:** Kyuseok Kim, Injin Ha, Eunok Kim, Kyunglee Kim

**Affiliations:** a Department of Ophthalmology, Otolaryngology and Dermatology, College of Korean Medicine, Kyung Hee University; b Korean Medicine Clinical Trial Center, Kyung Hee University Korean Medicine Hospital; c College of Korean Medicine, Kyung Hee University; d Department of Education, College of Education, Ewha Womans University, Seoul, Republic of Korea.

**Keywords:** acne vulgaris, biological biomarkers, diagnosis, metabolic biomarkers, patterns of acne

## Abstract

Acne is a multifactorial dermatosis, which is influenced not only by hormones but also by the biochemical relationship between them and the pilosebaceous unit. Inflammatory cytokines, chemokines, active oxygen, and zinc are known to be associated with the development of acne. Further, steroid metabolism is known as one of the important factors related to sebum secretion and comedone formation in acne. However, there is a lack of studies comparing these human biomarkers between healthy individuals and patients with acne. In particular, no study has investigated the relationship between human biomarkers and patterns of acne yet.

The purpose of this study is to investigate diagnostic human biomarkers in acne by comparing the biological and metabolic biomarkers between healthy individuals and patients with acne and identify the relationship between human biomarkers and patterns of acne.

This study is a protocol for a cross-sectional study. Forty healthy participants and 60 patients with acne will be recruited at 1 center. We will collect their blood samples and analyze the molecular biological and metabolic biomarkers (cytokines, chemokines, reactive oxygen species, corticotropin-releasing hormone, zinc, amino acid, 1-carbon metabolite, lipid metabolite, etc.). Further, we will administer questionnaires regarding their diet, sleep, stress, and other factors relating to acne and measure their skin elasticity.

The study protocol was approved by the Institutional Review Board of Oriental Medical Hospital at Kyung Hee Medical Center (KOMCIRB-161118-HR-062). Written informed consent will be obtained from all the participants. The trial was registered in the Clinical Research Information Service, Republic of Korea: KCT0002212.

This trial will provide evidence regarding diagnostic human biomarkers in acne and the relationship between the human biomarkers and patterns of acne.

## 1 Introduction

Acne vulgaris is a common chronic inflammatory dermatosis that manifests as papules, pustules, nodules, and cysts in the pilosebaceous units on the face, chest, and back.^[1,2]^ It is one of the most common diseases during adolescence, affecting more than 85% of adolescents.^[3]^ However, according to lifestyle factors, such as diet, occupation, and stress, the incidence of adult female acne, which starts after 25 years of age, is increasing.^[4]^ Acne is caused by multiple factors, such as increased sebum overproduction, abnormal cornification of the sebaceous follicular duct, colonization of the intrafollicular duct by *Propionibacterium acnes*, and inflammation.^[5,6]^ Genetic predispositions, hormonal abnormalities, immunological disorders, and psychological, environmental, and even iatrogenic factors may contribute to the formation of acne.^[7]^ Acne remains a rare disease in non-Westernized societies; however, the adoption of Western lifestyles increases the incidence of acne. Recent studies have reported that the Western diet is over-stimulated with the mammalian target of rapamycin complex-1 (mTORC1) signaling exaggerated cell growth and proliferation and mTORC1-mediated insulin resistance due to increased milk/dairy protein consumption with high glycemic loads and abundant amounts of branched chain amino acids,^[8,9]^ increased nutrient-sensitive kinase mTORC1 activity, and decreased nuclear levels of forkhead box class O1 transcription factor, which might aggravate or promote acne development.^[2,8]^

Although Western diet,^[10]^ stress,^[11]^ and smoking^[12]^ are factors that worsen acne, the exact etiology is still unknown. There is a lack of studies comparing these human biomarkers between healthy individuals and patients with acne, and no study has investigated the relationship between human biomarkers and patterns of acne yet.

The objective of this research is to determine the diagnostic specific human biomarkers in acne by comparing the molecular biological and metabolomics biomarkers between healthy control subjects and patients with acne, identify the relationship between human biomarkers and patterns of acne, and explore the correlation between biomarkers and various lifestyle factors affecting acne.

## 2 Methods and analysis

### 2.1 Aim of the study

The objective of this study is to discover novel specific biomarkers associated with the pathogenesis of acne by analyzing metabolomics/molecular biomarkers and skin biophysical parameters between healthy individuals and patients with acne, to compare the differences in these biomarkers according to the severity of acne, and to explore the correlation between such biomarkers and various factors, such as acne symptoms, dietary habit, stress level, sleep pattern, and menstrual pattern.

### 2.2 Design and registration

**Figure 1:**
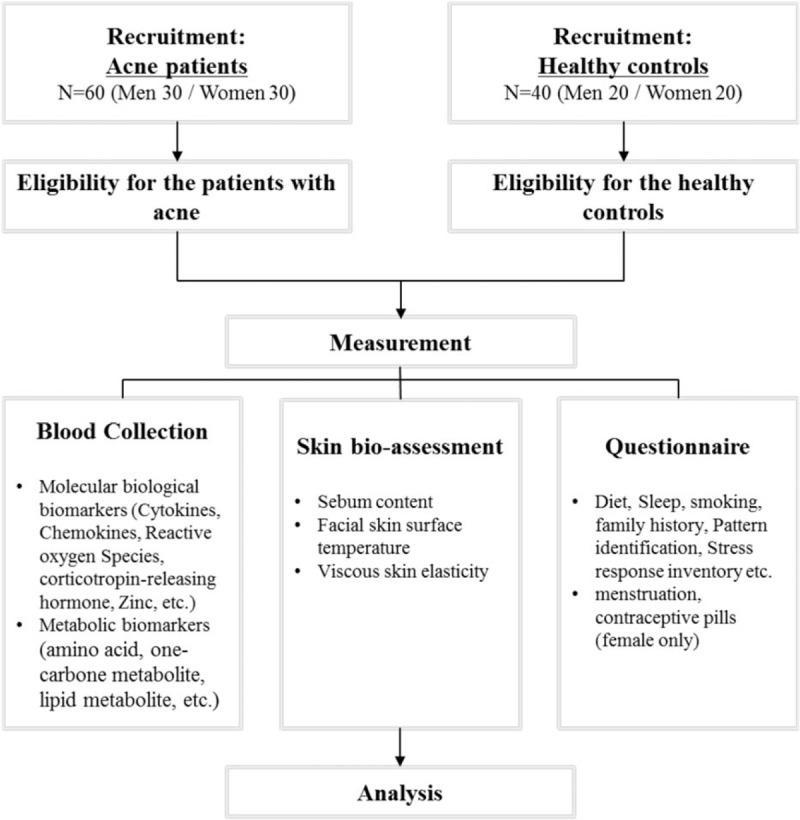
Flow of the study.

This cross-sectional study will be conducted at Kyung Hee University Korean Medicine Hospital from May 2017 to December 2017. Patients with acne and healthy volunteers will be recruited at the same time (Fig. 1). This trial was registered in the Clinical Research Information Service (CRIS), Republic of Korea: KCT0002212. The flow chart of this study is shown in Fig. 1. A completed Standard Protocol Items: Recommendations for Interventional Trials (SPIRIT) Checklist for the trial is available (see Additional file 1).

### 2.3 Participants and eligibility

#### 2.3.1 Inclusion criteria

##### 2.3.1.1 Participation of the population with acne vulgaris

Participants who will meet the following criteria will be included:

1. Age of 19 to 35 years.
2. Body mass index (BMI) of 18.5 to 23 kg/m^2^.
3. Acne vulgaris [20 individuals with Korean Acne Grading System (KAGS) Gr 1 or 2; 20 individuals with KAGS Gr 3 or 4; and 20 individuals with over KAGS Gr 5).
4. Voluntary participation in this clinical trial.

##### 2.3.1.2 Participation of the healthy controls

Participants who will meet the following criteria will be included:

1. Age of 19 to 35 years.
2. BMI of 18.5 to 23 kg/m^2^.
3. No evidence of acne.
4. (Only for women) regular menstrual cycle during the last 3 months.
5. No digestive-related uncomfortable symptoms in usual situations.
6. No abdominal muscle tension.
7. Other disorders that may affect the outcomes.
8. Voluntary participation in this clinical trial.

##### 2.3.1.3 Exclusion criteria

Participants who will meet any of the following will be excluded:

1. Use of oral steroids, oral contraceptives, oral vitamin A derivatives, antibiotics, or herbal medicines that may affect the test results during the last 4 weeks.
2. No fasting for more than 8 hours after 9:00 pm on the day before the visit.
3. Alcohol drinking within the last week.
4. Smoking.
5. Pregnancy or lactation.
6. Judgment that physical or mental testing is not appropriate for the clinical trial.
7. Participation in other clinical trials that may affect this trial.

### 2.4 Recruitment

Through advertisements, 60 patients with acne vulgaris and 40 healthy participants will be recruited at Kyung Hee University Korean Medicine Hospital. Advertisements will be posted on the notice boards of the hospital and local community centers. Consent will be obtained by the investigators with the principle of informed consent, which confirms the participants’ voluntarism. The participants will sign the consent form that would include the information on the use of their human-derived biomaterials, that is, blood sample.

### 2.5 Outcome measures

According to the schedule depicted on Table 1, we will use a series of outcome measures. There will be no difference in the outcomes measured between the patients with acne vulgaris and healthy subjects, except for the pattern identification related to acne symptoms. Facial photoshoot will be performed only on the participants with acne vulgaris. Five milliliters of whole blood will be collected in both patients with acne and healthy participants. The sera will be obtained from the whole blood via centrifugation 30 to 60 min after blood sampling.

**Table 1:**
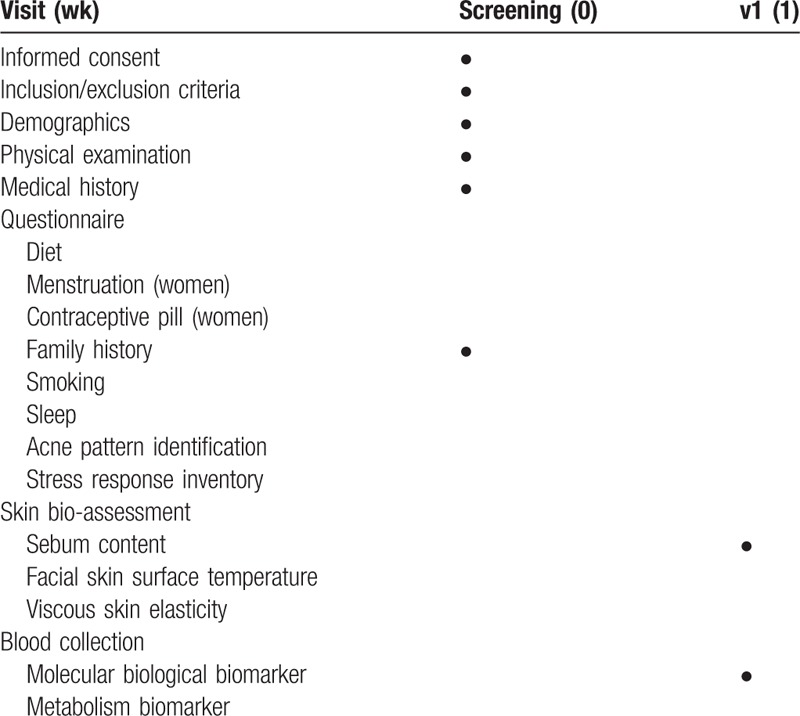
Study schedule for data collection and outcome measures.

#### 2.5.1 Questionnaire

The participants will complete a questionnaire that would include general sociodemographic information (e.g., age and sex), personal habits (e.g., smoking, alcohol consumption, sleep pattern, and dietary habits),^[4,13]^ anthropometric measurements (e.g., height, weight, and BMI), pregnancy, menstrual pattern and history, use of oral contraceptives, history of adolescence acne, history of acne in relatives, relevant comorbidities (e.g., polycystic ovary syndrome, hirsutism, type II diabetes, and thyroid disease), acne pattern identification,^[14]^ and stress level during the last month, which will be self-assessed in accordance with the stress response inventory.^[15]^

#### 2.5.2 Acne severity using the KAGS

Acne severity is defined in accordance with the KAGS shown in Table 2^[16]^ using photographs limited to the face to help with the assessments. A Lumix DMC-LX2 digital camera (Panasonic, Osaka, Japan) will be used to photograph the front and both sides of each patient's face, so that the inflammatory and noninflammatory lesions can be counted independently.

**Table 2:**
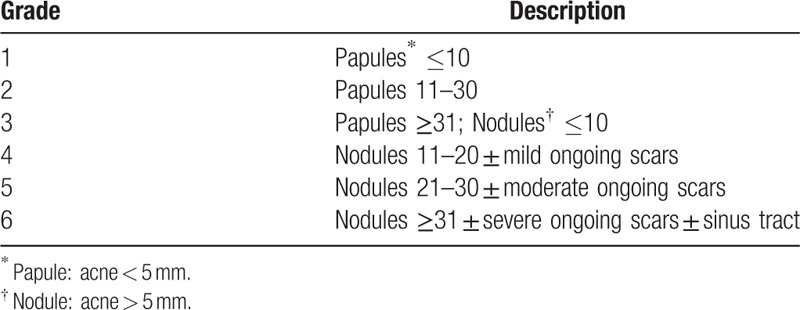
Korean acne grading system.

#### 2.5.3 Skin biophysical measurements

All biophysical measurements will be performed under strictly controlled conditions with a room temperature of 22 ± 2°C and a relative humidity of 50 ± 10% and after the resting period of ∼30 minutes. The participants will be instructed to follow certain guidelines before testing, that is, not to use cosmetics and not to wash the skin areas to be studied for ∼5 hours before the measurements. The measurements will be performed on the right and left upper cheeks, in the middle of both the eyebrows, and in the middle of the chin. All skin biophysical measurements will be conducted in accordance with the current guidelines.^[17]^ The skin biophysical parameters will be measured before and after the treatment using Sebumeter SM815, Skin-Thermometer ST500, and Reviscometer RVM600.^[18,19]^ Statistical analysis will be conducted with the average values of triplicate measurements for each parameter.

#### 2.5.4 Exploring biomarker with metabolomic profiling

This study will apply MS-based metabolomic profiling studies for the detection and identification of potential metabolic biomarkers for acne in the participants’ serum. The liquid chromatography-mass spectrometry system will consist of a Thermo Scientific Vanquish ultra-high-performance liquid chromatography (UHPLC) system (Thermo Fisher Scientific, Sunnyvale, CA) with a triple ToF 5600+ mass spectrometer system (Triple ToF MS) (SCIEX, Foster City, CA) for untargeted metabolite analysis. The UHPLC system with a QTRAP 3200TM (hybrid triple quadrupole/linear ion trap) mass spectrometry system (SCIEX, Foster City, CA). The analytical methods will be developed and optimized for targeted and untargeted analyses of metabolites, such as steroidal hormones (dehydroepiandrosterone, androstenedione, testosterone, dihydrotestosterone, estrone, and estradiol), fatty acids (oleic acid; cis, elaidic acid; trans, palmitic acid; saturated), and endogenous small metabolites closely related to energy metabolism and lipid/steroid biosynthesis. Statistical techniques will be used to investigate the particular metabolic changes and entire network of the metabolic pathway profile.

#### 2.5.5 Exploring biomarker with biological profiling

The whole blood, which will be collected in serum tubes, will be allowed to clot by leaving it at room temperature for 30 minutes. The clot will be removed by centrifuging at 3000 rpm for 10 minutes. All the serum samples will be stored at –70°C until further analysis. The concentrations of the cytokines (interleukin [IL-1α], IL-1β, IL-12β, IL-15, IL-6, and tumor necrosis factor-α) and chemokines (monocyte chemoattractant protein-1 and IL-8) in the serum will be determined using a bead-based multiplex assay for luminex platform (R&D Systems, Minneapolis, MN) according to the manufacturer's instructions and measured using a Luminex 200 system (Luminex Corporation, Austin, TX). Each serum sample will be run in duplicates and averaged to calculate the concentrations. The Enzyme-Linked Immunosorbent Assay kits that will be used to analyze the serum calcium, zinc, and ROS will be purchased from Abcamb (Cambridge, UK). Each product will be used following the instructions provided by the manufacturer. The absorbance will be measured at 450 nm using the microplate reader (Versa Max) and analyzed using the SoftMax pro software (Molecular Devices).

#### 2.5.6 Standard operating procedures for quality assurance

We have established detailed standard operating procedures for this clinical trial, educated all practitioners, and set qualification standards to make sure the participants in accordance with the trial protocol. Practitioners and medical equipment managers attended a 2-day training workshop on the trial procedures and were educated on the methods of obtaining the photograph of the patients’ face and of using the KAGS, Sebumeter SM815, Skin-Thermometer ST500, and Reviscometer RVM600. A written protocol and standardized recording documents will be provided.

### 2.6 Data collection, management, and quality control

To maintain the quality of this trial, and data management including data collection, validation, and completion will be conducted by the Kyung Hee Korean Medicine-Clinical Trial Center (K-CTC) in Seoul, Korea. To ensure that outcome assessments are of a high standard in accordance with the trial protocol, the investigator and the assistants will attend a 6-hour training workshop prior to the initiation of the trial. The investigator and the assistants will also be provided with a written protocol and standard operating procedure documents. All the data will be checked regularly by clinical trial coordinators from K-CTC.

### 2.7 Sample size

The current study is a preliminary cross-sectional trial with the aims of discovering novel specific biomarkers in acne by investigating metabolomics/molecular biomarkers between healthy individuals and patients with acne, comparing the differences in these biomarkers according to the severity of acne, and exploring the correlation between the biomarkers and acne symptoms. In other words, this study is, to the best of our knowledge, the first of its kind and not intended to validate affirmatively the effects of certain interventions on acne, but rather to compare the biomarkers of patients with acne with those of healthy individuals. If the aims of the pilot study do not include any hypothesis tests, the power level of a test procedure is not a valid consideration for sample size.^[20]^ Moreover, in the metabolomics study design, a sample size of 3 to 5 per group may give useful preliminary data that can be used to design more elaborate and/or targeted metabolomics studies and the number of sample size for a metabolomics experiment depends on the biological variability associated with the system being studied compared with the analytical variability of the analytical system.^[21]^ Therefore, in this exploratory study, the number of patients to be included will be set to 40 healthy subjects (20 men and 20 women) and 60 patients with acne (30 men and 30 women) which enables the assessment of the difference in metabolomics/molecular biomarkers between healthy individuals and patients with acne including a respective 95% confidence interval, with sufficient precision according to the size of the metabolite kit with expert opinions in metabolomics, molecular biology, dermatology, and statistics.

### 2.8 Statistical methods

Data will be displayed as means ± standard deviations for continuous data or n (%) for categorical data. Statistical analyses will be conducted with a 95% confidence interval using the SPSS 21.0 (IBM SPSS Statistics, New York, NY). An independent *t* test will be performed for the comparison of specific biomarkers associated with the pathogenesis of acne by analyzing metabolomics/molecular biomarkers and skin biophysical parameters between the healthy subjects and participants with acne vulgaris. One-way analysis of variance will be performed for the comparison of the differences in these biomarkers among 3 groups (mild, Gr 1-2; moderate, Gr 3-4; severe, Gr 5-6), which we will classify in accordance with the severity of acne using the KAGS. The correlation between the biomarkers and various factors, such as acne symptoms, dietary habit, stress level, sleep pattern, and menstrual pattern, will be examined using the Pearson correlation test. The mean value of a variable will be used in place of the missing data value for that same variable.

### 2.9 Ethics and dissemination

The study protocol version 1.2 was approved by the Institutional Review Board of Oriental Medical Hospital at Kyung Hee Medical Center (KOMCIRB-161118-HR-062). The approved protocol was registered with in the CRIS, Republic of Korea: KCT0002212. Any modification to the protocol will be reapproved by the IRBs, documented in the online registry of CRIS, and reflected in the explanation for the participants. Each participant will be provided with information regarding the study protocol. Written informed consent will be obtained from each patient. The privacy of all participants will be protected. Personal medical records will be reviewed by investigators, who will promise to keep the content confidential. Data anonymity will be used in the data management. Finally, the data of all subjects will be kept in the document storage room of Kyung Hee K-CTC. It will be performed in accordance with the standards of the International Committee on Harmonization on Good Clinical Practice and the revised version of the Declaration of Helsinki principles.

The result of this study will be published in peer-reviewed journals or academic conferences.

### 2.10 Protocol amendment

Any modifications to the protocol which have substantial effects on the conduct of the study, potential benefits to the patient or impact on patient safety, including changes to eligibility criteria, patient population, sample size, study objectives, study design, and study procedures will require a formal amendment to the protocol. Such amendment will be sent to the Ethics committee for further approval before implementation. Administrative changes of the protocol defined as minor corrections having no effect on the way the study is to be conducted will be documented and the Ethics committee may also be notified of them.

## 3 Discussion

Although accumulating evidence underlines the role of Western diet, stress, and smoking in the pathogenesis of acne, the pathogenesis and aggravating factors of acne are not yet fully elucidated. Therefore, the metabolomics/molecular biomarkers could be an issue when discussing acne management with patients.

This will be the first protocol that would discover new diagnostic biomarkers in acne by analyzing metabolomics/molecular biomarkers and skin biophysical parameters between healthy individuals and patients with acne, compare the differences in these biomarkers according to the severity of acne, and explore the correlation between these biomarkers and various factors. In this study, we will analyze the molecular biomarkers, including zinc, calcium, and ROS, as well as existing known inflammatory cytokines and chemokines, and targeted and untargeted metabolite biomarkers, such as steroidal hormones, fatty acids, and endogenous small metabolites closely related to energy metabolism and lipid/steroid biosynthesis in terms of systems biology. We will also use the skin biophysical measurement devices Sebumeter SM815, Skin-Thermometer ST500, and Reviscometer RVM600 and questionnaires that would include general sociodemographic information, personal habits, anthropometric measurements, pregnancy, menstrual pattern and history, use of oral contraceptives, history of adolescence acne, history of acne in relatives, relevant comorbidities, acne pattern identification, and stress level.

Several methodological limitations within this study warrant further discussion. First, only young adults will be studied, and the sample size was decided in accordance with the size of the metabolite kit with expert opinions in metabolomics, molecular biology, dermatology, and statistics and not a power analysis. Second, diet tracking will not be performed. Third, this study has a cross-sectional design; because of the nature of this study design, interpretations of the results will be limited.

Despite these limitations, this study could be helpful in providing new diagnostic human biomarkers in acne and correlation between human biomarkers and patterns of acne. The findings of this trial may have important implications for the new approach of personal individualized therapy by identifying and differentiating the treatment methods of acne according to the biological and metabolic biomarkers based on human blood samples and patterns of acne.

## References

[1] Burris J Rietkerk W Woolf K Acne: the role of medical nutrition therapy. J Acad Nutr Diet 2013;113:416–30. 23438493 10.1016/j.jand.2012.11.016

[2] Çerman AA Aktaş E Altunay İK Dietary glycemic factors, insulin resistance, and adiponectin levels in acne vulgaris. J Am Acad Dermatol 2016;75:155–62. 27061046 10.1016/j.jaad.2016.02.1220

[3] Melnik BC Evidence for acne-promoting effects of milk and other insulinotropic dairy products. Nestle Nutr Workshop Ser Pediatr Program 2011;67:131–45. 10.1159/000325580 21335995

[4] Di Landro A Cazzaniga S Cusano F Group for Epidemiologic Research in Dermatology Acne Study Group. Adult female acne and associated risk factors: results of a multicenter case-control study in Italy. J Am Acad Dermatol 2016;75:1134–41. 27542588 10.1016/j.jaad.2016.06.060

[5] Rahaman SM De D Handa S Association of insulin-like growth factor (IGF)-1 gene polymorphisms with plasma levels of IGF-1 and acne severity. J Am Acad Dermatol 2016;75:768–73. 27476104 10.1016/j.jaad.2016.05.019

[6] Napolitano M Megna M Monfrecola G Insulin resistance and skin diseases. Scientific World J 2015;2015:479354. 10.1155/2015/479354 PMC4419263 25977937

[7] Kucharska A Szmurło A Sińska B Significance of diet in treated and untreated acne vulgaris. Postepy Dermatol Alergol 2016;33:81–6. 27279815 10.5114/ada.2016.59146 PMC4884775

[8] Melnik BC John SM Plewig G Acne: risk indicator for increased body mass index and insulin resistance. Acta Derm Venereol 2013;93:644–9. 23975508 10.2340/00015555-1677

[9] Bowe WP Joshi SS Shalita AR Diet and acne. J Am Acad Dermatol 2010;63:124–41. 20338665 10.1016/j.jaad.2009.07.043

[10] Ju Q Tao T Hu T Sex hormones and acne. Clin Dermatol 2017;35:130–7. 28274349 10.1016/j.clindermatol.2016.10.004

[11] Bhate K Williams HC Epidemiology of acne vulgaris. Br J Dermatol 2013;168:474–85. 23210645 10.1111/bjd.12149

[12] Capitanio B Sinagra JL Ottaviani M Acne and smoking. Dermatoendocrinol 2009;1:129–35. 20436880 10.4161/derm.1.3.9638 PMC2835905

[13] Kulkarni M Keny D Potey AV A cross-sectional study to assess the incompatible dietary behavior of patients suffering from skin diseases: a pilot study. J Ayurveda Integr Med 2016;7:113–8. 27450757 10.1016/j.jaim.2016.06.001 PMC4969308

[14] Shin J Jeong W Moon Y An expert survey for developing the pattern diagnosis instrument of acne. J Korean Med Ophthalmol Otolaryngol Dermatol 2015;28:23–32.

[15] Koh KB Park JK Kim CH Development of the stress response inventory and its application in clinical practice. Psychosomatic Med 2001;63:668–78. 10.1097/00006842-200107000-00020 11485121

[16] Sung K Rho Y Choi E Korean acne grading system. Korean J dermatol 2004;42:1241–7.

[17] Trojahn C Dobos G Lichterfeld A Characterizing facial skin ageing in humans: disentangling extrinsic from intrinsic biological phenomena. Biomed Res Int 2015;2015:318586. 25767806 10.1155/2015/318586 PMC4341846

[18] Rodrigues L EEMCO guidance to the in vivo assessment of tensile functional properties of the skin. Part 2: instrumentation and test modes. Skin Pharmacol Appl Skin Physiol 2001;14:52–67. 10.1159/000056334 11174091

[19] Firooz A Sadr B Babakoohi S Variation of biophysical parameters of the skin with age, gender, and body region. Scientific World J 2012;2012:386936. 10.1100/2012/386936 PMC3317612 22536139

[20] Moore CG Carter RE Nietert PJ Recommendations for planning pilot studies in clinical and translational research. Clin Transl Sci 2011;4:332–7. 22029804 10.1111/j.1752-8062.2011.00347.x PMC3203750

[21] Barnes S Benton HP Casazza K Training in metabolomics research. I. Designing the experiment, collecting and extracting samples and generating metabolomics data. J Mass Spectrom 2016;51:461–75. 27434804 10.1002/jms.3782 PMC4964969

